# Differences in race history by distance of recreational endurance runners from The NURMI Study (Step 2)

**DOI:** 10.1038/s41598-023-45055-w

**Published:** 2023-10-23

**Authors:** Beat Knechtle, Derrick Tanous, Mabliny Thuany, Mohamad Motevalli, Gerold Wirnitzer, Claus Leitzmann, Katja Weiss, Thomas Rosemann, Katharina Wirnitzer

**Affiliations:** 1 Institute of Primary Care, University of Zurich, 8000 Zurich, Switzerland; 2 Medbase St. Gallen Am Vadianplatz, Vadianstrasse 26, 9000 St. Gallen, Switzerland; 3 Department of Sport Science, University of Innsbruck, 6020 Innsbruck, Austria; 4 Department of Research and Development in Teacher Education, University College of Teacher Education, Tyrol, 6020 Innsbruck, Austria; 5 Faculty of Sports, University of Porto, Porto, Portugal; 6 AdventureV & change2V, 6135 Stans, Austria; 7 Institute of Nutrition, University of Gießen, 35390 Gießen, Germany; 8 Research Center Medical Humanities, Leopold-Franzens University of Innsbruck, 6020 Innsbruck, Austria

**Keywords:** Metabolism, Epidemiology

## Abstract

Few studies were developed to understand the relationship between running characteristics and motivation. The purpose of this study was to assess the relationship between running event history, running experience, and best race performances in recreational distance runners. We used a web survey to obtain information regarding running experience, racing history, and periodization training routines/exercise habits, including weekly volumes and daily mileage and duration across periods and conditions. Associations between variables were conducted with the Chi-square test (χ^2^; nominal scale) and Wilcoxon test. Multiple linear regression analysis and multivariate linear regression were performed. Concerning the participants’ motive for exercising, a significant difference was identified between the race distance subgroups (p < 0.001), where 58% of M/UM runners exercised for performance (n = 38) and 64% of HM runners (n = 57) and 57% of 10 km runners (n = 52) exercised for recreational purposes. A significant difference was found in the number of years of running completed without taking a break (p = 0.004), with marathoners/ultramarathoners reporting the most years. Runners competing in different race distances such as 10 km, half-marathon, marathon, and ultra-marathon presented differences in training background and habits according to the distance of preference.

## Introduction

Running is a global market, with an increase in the participation of athletes in running events and the number of events over the last year worldwide^1–3^. In the European context, a range of 5% to 31% rate of participation was shown between different countries^4^. In a scientific context, this growth was associated with a higher interest for understanding runners’ profiles, behaviors, and training habits^5,6^. The runner’s profile was previously studied in different contexts, including differences in economic level^7,8^, the profiles consumption and use of sports watches^9^, training characteristics^5,10^, nutritional behaviors^11–13^, and health outcomes^14^.

As a social phenomenon, and with the potential to improve general physical (i.e., lower risks of all-cause and cardiovascular mortality)^15^ and mental health (i.e., well-being, self-confidence), running is also related to social cohesion^16^ and used as a potential strategy to improve physical activity levels in an epidemiological context^17^. In this way, the reasons to start running and to be engaged in running training were also investigated previously^18^. For non-professional runners, motivational differences were shown in athletes competing in different race distances^19–22^. For runners in 5 km, fun and health were the most important factors for training^23^, while ultra-marathoners had higher scores in affiliation, life meaning, and lower body weight concerns^24^.

Based on previous studies, a body of evidence is available regarding motivational characteristics and runners’ profiles^19,25,26^. However, few studies were developed to understand the relationship between running background and motivation^27^. Understanding the motives and habits considering training and competing that enable non-professional runners to be engaged in physical exercise is an important feature to provide support and to understand why people are or not engaged in running, as well as to develop strategies to maintain the training commitment.

Therefore, this is the first exploratory investigation to assess the aspects of motivation, education, training, previous experience, and performance in different running groups such as 10 km, half-marathon (HM), and marathon (M)/ultramarathon (UM) recreational distance runners. Based on previous studies^28–30^, it is assumed that there would be differences in these aspects in recreational endurance runners of different distances (10 km, HM, M/UM)^10^.

## Materials and methods

**Figure 1:**
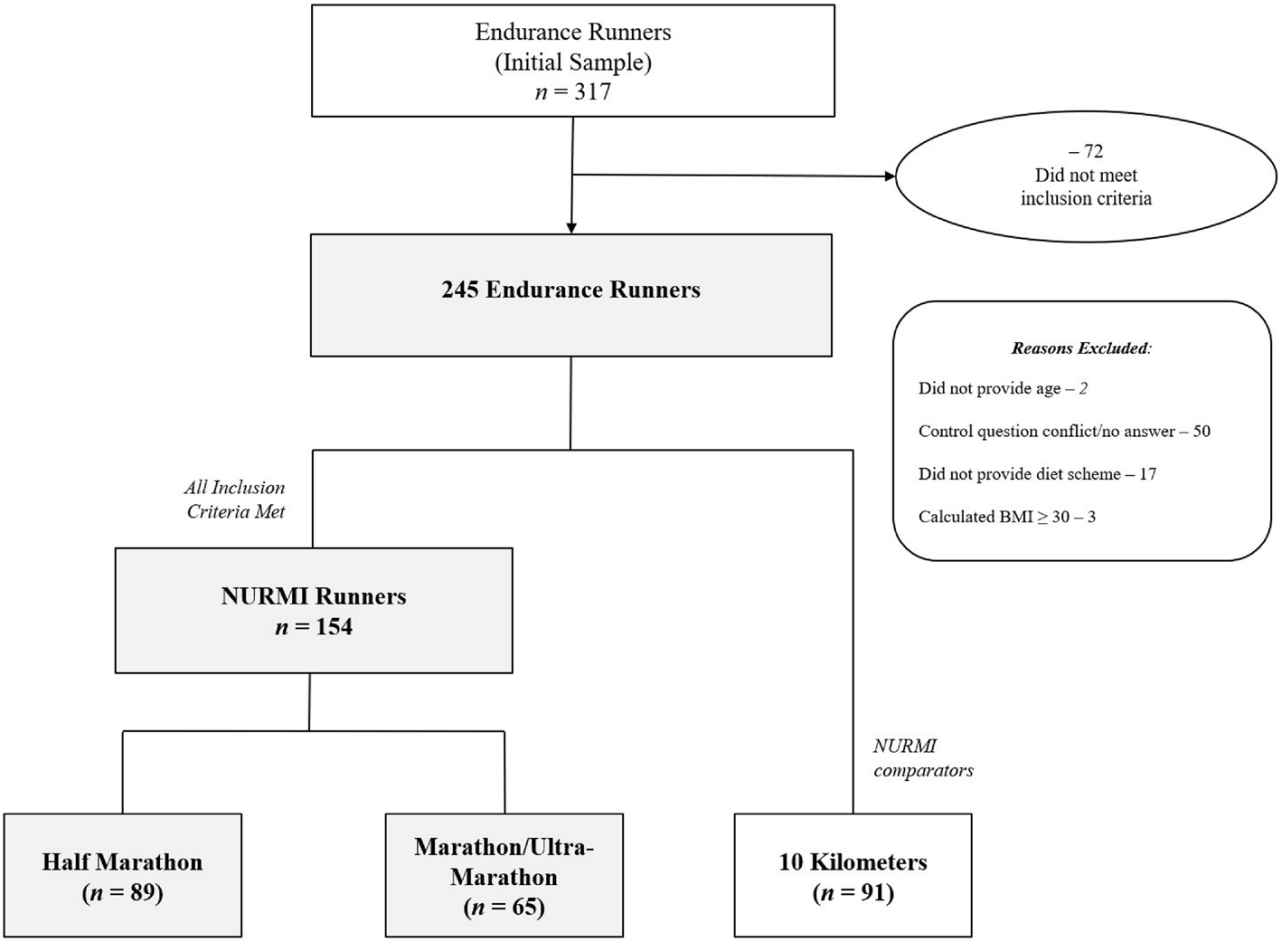
Enrollment and Categorization of Participants by Race Distance.

Please see the subsequent description of the methodology for the complete profile for this investigation (Part A of the arrangement)^31^, as well as previous publications^10,32–34^. Following a protocol^35^, the Nutrition and Running High Mileage (NURMI) Study has been approved by the ethics board of St. Gallen, Switzerland on the 6^th^ of May in 2015 (EKSG 14/145) with a retrospective trial registration (number: ISRCTN73074080). It was required that the participants provided informed consent before taking part in the NURMI Study. For the participants’ recruitment and study procedures, the responsive reader is kindly referred to Part A of the arrangement publication^31^. Figure 1 shows the enrollment and categorization of participants, and their characteristics are shown in Table 1.

**Table 1:** Runner characteristics, including motive to race, experience, and history displayed race distance.

	Total 100% (245)	10 km37% (91)	HM 36% (89)	M/UM 27% (65)	Statistics
Age (Years)	39 (IQR 17)	37 (IQR 18)	37 (IQR 18)	44 (IQR 17)	F_(2,242)_ = 4.87 p = 0.008
BMI (kg/m^2^)	21.7 (IQR 3.5)	21.3 (IQR 3.94)	22 (IQR 3.28)	22.2 (IQR 3.25)	F_(2,242)_ = 1.22 p = 0.296
Civil status					
Single	27% (66)	26% (24)	31% (28)	22% (14)	χ^2^_(4)_ = 1.95 p = 0.744
With spouse/married	67% (164)	67% (61)	63% (56)	72% (47)	
Separated/divorce	6% (15)	7% (6)	6% (5)	6% (4)	
Motive to race					
Leisure	46% (106)	41% (36)	47% (41)	51% (29)	χ^2^_(2)_ = 1.34 p = 0.512
Performance	54% (125)	59% (51)	53% (46)	49% (28)	
Favorite season of racing					
Winter	< 1% (2)	1% (1)	1% (1)	/	χ^2^_(6)_ = 9.04 p = 0.171
Spring	46% (106)	36% (31)	55% (48)	47% (27)	
Summer	23% (52)	28% (24)	15% (13)	26% (15)	
Autumn	31% (71)	36% (31)	29% (25)	26% (15)	
Running experience (years)	7 (IQR 7)	5 (IQR 8)	7 (IQR 6)	8 (IQR 9)	F_(2, 241)_ = 5.77 p = 0.004
First event age (years)					
10 km	30 (IQR 16)	30 (IQR 17)	28 (IQR 15)	33 (IQR 17)	F_(2, 151)_ = 0.69 p = 0.502 F_(2, 216)_ = 1.17 p = 0.313 F_(2, 135)_ = 0.18 p = 0.836 F_(2, 239)_ = 1.77 p = 0.172
HM	32 (IQR 16)	33 (IQR 15)	30 (IQR 18)	35 (IQR 13)	
M	35 (IQR 13)	33 (IQR 15)	34 (IQR 17)	35 (IQR 12)	
Total	30 (IQR 16)	30 (IQR 17)	28 (IQR 18)	34 (IQR 13)	
First event					
10 km	65% (157)	81% (74)	59% (52)	48% (31)	χ^2^_(4)_ = 46.24 p < 0.001
HM	27% (65)	18% (16)	38% (33)	25% (16)	
M	9% (21)	1% (1)	3% (3)	27% (17)	
Total races completed	8 (IQR 11)	7 (IQR 11)	6 (IQR 11)	10 (IQR 11)	F_(2, 242)_ = 2.90 p = 0.057
Ratio of HM/M to total races	40 (IQR 50)	20 (IQR 35)	48 (IQR 43)	53 (IQR 49)	F_(2, 242)_ = 18.44 p < 0.001
Completion of planned events (previous 2 years)					
HM	2 (IQR 3)	1 (IQR 2)	3 (IQR 4)	2 (IQR 3)	F_(2, 242)_ = 7.04 p = 0.001 F_(2, 242)_ = 75.19 p < 0.001 F_(2, 242)_ = 28.84 p < 0.001
M	1 (IQR 2)	0 (IQR 1)	0 (IQR 1)	2 (IQR 2)	
UM	0 (IQR 0)	0 (IQR 0)	0 (IQR 0)	0 (IQR 1)	

*Note* Results are presented as percentage (%), total numbers, and median (IQR). χ^2^ statistic calculated by Pearson’s Chi-squared test and *F* statistic calculated by Kruskal–Wallis test. 10 km 10 km. *HM* half-marathon. *M/UM* marathon/ultra-marathon.

### Measures

Race performances, training routines, and exercise habits of active distance runners were expressed using the following parameters: running experience (total number of years of running fully completed without taking a break); racing history (overall number of completed races, ratio of HM/M events to total races, age at time of the first running event, the first race distance completed: 10 km, HM, M, best HM/M times, the number of planned races completed in the previous two years: HM/M/UM); periodization training routines/exercise habits, including weekly volumes (number of running sessions, and breadth of training in km and hours) and daily mileage and duration across periods and conditions. Running performance was related to best finishing HM and M time based on a normalized aggregate mean transformed to an index (ranging 0–100). The latent variable of running history was derived by both factors: (1) “running-experience” (by pooled items: “age.first.running event”, “age.run”, “age.first.half-marathon”, “age.first.marathon”) and (2) “racing-experience” (by pooled items: “years.running”, “completed.half-marathon.number”, “completed.marathon.number”), which were defined by specific items that were based on manifest variables.

As running experience (e.g., years of running fully completed, age at the first race event, total number of races completed) is dependent upon age, the respective items were operationalized with age (e.g., age-related years of running, age-related number of completed races over half-marathon distance). Based on this, the respective items (e.g., age-related beginning of running, first marathon race completed) were centered by median values, and were z-transformed creating a new scale through summarizing the respective items (e.g., years of running fully completed, completed races over specified distances). From this the values were categorized with the latent factors “running-experience” and “racing-experience” into low (values below − 1), medium (values ranging from − 1 to + 1), and high (values higher + 1). A principal component analysis (PCA as heuristic approach) was performed to identify the respective factors. The PCA was justified by sufficient high correlations (0.79 by the Kaiser–Meyer–Olkin-Kriterium, and p < 0.001 by the Bartlett-Test as highly significant) to derive the extraction of two factors. The “Eigen”-Wert > 1 (declaration of 73.4% of total variance of both the latent factors) was defined to justify to model two latent factors: “*running-experience*” (from items: “age.first.running event”, “age.run”, “age.first.half-marathon”, “age.first.marathon”) and “*racing-experience*” (from items: “years.running”, “completed.half-marathon.number”, “completed.marathon.number”).

### Statistical analysis

The statistical analyses were all performed with R software (version 3.6.2 Core Team 2019; R Foundation for Statistical Computing; Vienna, Austria). The exploratory analysis was performed with descriptive statistics, including median with interquartile range (IQR) and mean with standard deviation (SD). PCA was used for identifying the latent factors.Significant differences in running and racing activity (experience, training, racing, etc.) between race distance subgroups were calculated with a non-parametric test. Associations between variables were conducted with Chi-square test (χ^2^; nominal scale) and Wilcoxon test (ordinal and metric scale) have been approximated by using F distributions and ordinary least squares. Multiple linear regression analysis and multivariate linear regression were performed to test the differences in performance, health, and leisure motivations based on race distance subgroups. The regression results are displayed as effect plots with a 95% confidence interval (95%-CI). The level of statistical significance was set at p ≤ 0.05.

### Institutional review board

The study protocol is available online via https://springerplus.springeropen.com/articles/10.1186/s40064-016-2126-4 and was approved by the ethics board of St. Gallen, Switzerland on May 6, 2015 (EKSG 14/145). The study was conduct-ed in accordance with the ethical standards of the institutional review board, medical professional codex, and with the 1964 Helsinki declaration and its later amendments as of 1996, the Data Security Laws, and good clinical practice guidelines. Study participation was voluntary and could be canceled at any time without the provision of reasons or negative consequences. In-formed consent was obtained from all individual participants included in the study considering the data collected, used, and analyzed exclusively and only in the context of the NURMI Study for scientific publication.

## Results

The total sample included 317 runners of various long distances who finished and submitted the questionnaire. A sum of 72 participants were excluded due to failing to meet the inclusion criteria following data clearance. The final sample was comprised of 245 runners (10 km: n = 91; NURMI runners: HM: n = 89; M/UM: n = 65), including 104 males and 141 females. Together the participants had a BMI of 21.7 kg/m^2^ (body weight of 65 kg, height of 1.7 m) and were aged 39 years. Regarding the participants’ nationalities, 72% came from Germany (n = 177), 18% were from Austria (n = 44), and 9% were from Switzerland (n = 13) or another country (n = 11).

Significant differences were observed across the race distance subgroups for height (p = 0.007), body weight (p = 0.007), and age (p = 0.008) with the M/UM participants being taller (1.8 m, IQR 0.1), heavier (67.5 kg, IQR 17.5), and older (44 years, IQR 17). No significant difference was observed across the race distance subgroups for BMI (p = 0.296) or for civil status (p = 0.744), most participants were married or living with their spouse (67%; n = 144) or single (27%; n = 66). No significant differences were found for race distance subgroups regarding the participants’ educational background (p = 0.177): 1 (< 1%) had no qualification, 53 (22%) held an A-Levels (or similar degree), 83 (34%) held an upper secondary school/technical education degree, 83 (34%) held a university degree (or possibly higher), and 25 (10%) did not answer. Concerning the participants’ motive for exercising, a significant difference was identified between the race distance subgroups (p < 0.001), where 58% of M/UM runners exercised for performance (n = 38) and 64% of HM runners (n = 57) and 57% of 10 km runners (n = 52) exercised for recreational purposes. The participants’ characteristics, including their motive to race and running experiences are shown in Table 1 based on their self-reported race distances. In Part A, additional details on the total sample’s profile and the race distance-specific subgroups are provided^31^.

No significant differences were found across the race distance subgroups for the motive to race (p = 0.512) or the current motive to run (p = 0.583); performance was the most frequently reported racing motive (54%; n = 125) among the whole sample. No significant difference was observed for the favorite race season (p = 0.171); springtime was the most favored season for racing for all participants (46%; n = 106), while winter was the least favored (< 1%; n = 2). A significant difference was found in the number of years of running completed (consecutively or inconsecutively) without taking a break (p = 0.004), with M/UM runners reporting the most years (8; IQR 9) and 10 km runners reporting the least (7 IQR 11). Regarding racing history, significant differences between race distance subgroups were found in (i) the ratio of completed HM/M events to the total races, where M/UM runners had the highest reports (53; IQR 49; p < 0.001); (ii) the first race distance, where most 10 km (81%; n = 74) and HM (59%; n = 52) runners first completed a 10 km race (p < 0.001); (iii) the best time for a HM race, where M/UM runners were the fastest on average (99 min ± 13; p < 0.001); (iv) the best time for a M race, where M/UM runners were the fastest on average (218 min ± 34; p = 0.029); (v) the completion of HM (p = 0.001), M (p < 0.001), and UM (p < 0.001) races in the previous two years, where HM runners completed the most HM races (3; IQR 4) and M/UM runners completed the most M (2; IQR 2) and UM (0; IQR 1) races. No significant differences in racing history between race distance subgroups were identified in overall completed races (p = 0.057), first event age in total (p = 0.172), or regardless of 10 km (p = 0.502), HM (p = 0.313), or M distance (p = 0.836).

**Figure 2:**
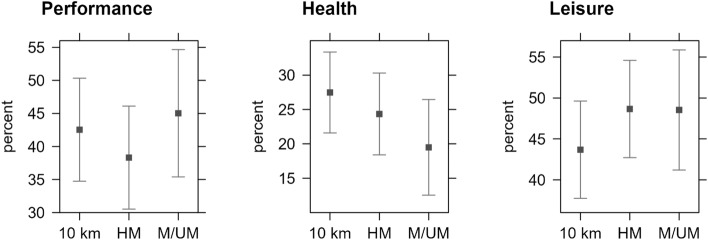
Effect plots displaying 95%-CI average between 10 km, HM, and M/UM subgroups in exercise/running/racing motives (n = 231). *Note* 95%-CIs were computed using the multivariate regression analyses (Wald approximation).

Non-significant relationships were identified in multivariate linear regression, as seen in Fig. 2, between (i) the motives of performance, the 10 km subgroup, and the HM subgroup (b = − 4.21; 95% CI [− 15.2 to 6.81]; p > 0.05) or the M/UM subgroup (b = 2.5; 95% CI [− 9.89 to 14.9]; p > 0.05); (ii) the motives of health, the 10 km subgroup, and the HM subgroup (b = − 3.07; 95% CI [− 11.5 to 5.39]; p > 0.05) or the M/UM subgroup (b = − 7.9; 95% CI [− 17.4 to 1.6]; p > 0.05); (iii) the motives of leisure, the 10 km subgroup, and the HM subgroup (b = 4.98; 95% CI [− 3.42 to 13.4]; p > 0.05) or the M/UM subgroup (b = 4.86; 95% CI [− 4.59 to 14.3]; p > 0.05).

Multivariate linear regression was performed and the following confounders were included within different models to predict the best HM and M race time between 10 km and HM or M/UM race distance subgroups: (a) years of running history and age at the first running event, which determined 21% of variance (adjusted R^2^ = 0.21) and a significant difference was identified for M/UM runners (*b* = 10.9; 95% CI [1.74–20]; p < 0.05) but not for HM runners (*b* = − 5.72; 95% CI [− 14.1 to 2.65]; p > 0.05); (b) training routines and exercise habits (including preparation condition 3, preparation condition 4, weekly kilometers of preparation condition 1, professional support, and the training extent for main race in months), which determined 22% of variance (adjusted R^2^ = 0.22) and no significant difference for HM (*b* = − 6; 95% CI [− 14.6 to 2.65]; p > 0.05) or M/UM (*b* = 0.679; 95% CI [− 9.09 to 10.5]; p > 0.05) race distance groups; (c) racing history (total races completed, the ratio of HM/M events to total events, HM races completed, and M races completed), which determined 16% variance (adjusted R^2^ = 0.16) and no significant difference for HM (*b* = − 6.26; 95% CI [− 15 to 2.45]; p > 0.05) or M/UM (*b* = 7.6; 95% CI [− 3.47 to 18.7]; p > 0.05) race distance subgroups. In Table 2, multiple linear regression analyses are provided.

**Table 2:** Multiple linear regression analyses on running experience, training routines and exercise habits, and racing history.

Adjusted r^2^	*Model 1* 0.21			*Model 2* 0.22			*Model 3* 0.16		
	*b*	*95%-CI*	*p*	*b*	*95%-CI*	*P*	*b*	*95%-CI*	*p*
*Intercept*	68.8	56.2–81.5	< 0.001						
Years of running experience	0.765	− 0.28–1.25	< 0.01						
First event age	− 0.97	− 1.3 to − 0.64	< 0.001						
HM Subgroup	− 5.72	− 14.1 to 2.65	> 0.05						
M/UM Subgroup	10.9	1.74–20	< 0.05						
*Intercept*				32	19.4–44.7	< 0.001			
Preparation condition 3				5.92	1.85–9.98	< 0.01			
Preparation condition 4				− 2.14	− 5.98 to 1.7	> 0.05			
Prep Condition 1: Weekly km				0.274	0.1–0.45	< 0.01			
Professional support				10.6	0.17–21.1	< 0.05			
Training extent for main race				− 1.2	− 3.17 to 0.77	> 0.05			
HM subgroup				− 6	− 14.6 to 2.65	> 0.05			
M/UM subgroup				0.679	− 9.09 to 10.5	> 0.05			
*Intercept*							39.4	28.7–50.1	< 0.001
Races completed in total							0.558	0.05–1.07	< 0.05
Ratio of HM/M to total races							− 0.0746	− 0.22 to 0.08	> 0.05
HM races completed							0.962	− 0.94 to 2.86	< 0.05
M races completed							0.634	− 1.7 to 2.97	> 0.05
HM subgroup							−6 .26	− 15 to 2.45	> 0.05
M/UM subgroup							7.6	− 3.47 to 18.7	> 0.05

*Note b* = estimate (marginal effects), *CI* confidence interval, *HM* half-marathon, *M* marathon, *UM* ultra-marathon.

## Discussion

This study was the first exploratory investigation aiming to analyze running event history, running experience, and best race performance between 10 km, HM, and M/UM recreational runners. The most important findings were (i) the runners had a similar BMI regardless of race distance subgroup even though M/UM runners were the tallest participants and weighed the most; (ii) no difference was found across race distance subgroups in the motive to race or for the linked motives (i.e. exercise motive, original motive to run, present motive to run, motive to race); (iii) M/UM runners tallied significantly more years of fully active running experience and completed significantly more of their planned marathon and ultra-marathon races in the previous two years; (iv) significant differences between the race distance subgroups in best time performances, where M/UM runners were fastest on average to complete HM and M events, however, when analyzing best time performances with an index and applying confounders (training routines and exercise habits; racing history) in multivariate linear regression analyses, no significant differences in performance were found between subgroups; (v) M/UM runners remained fastest on average to complete HM and M events when considering the confounders of running experience in years fully active in running without break and the participants’ age at their first running event. Thus, this exploratory investigation upholds the assumption that there is a difference in the best race performances considering time to finish between recreational endurance runners of different distances (10 km, HM, M/UM).

### Differences in anthropometry and age across groups

We found that the runners had a similar BMI regardless of race distance subgroup, even though M/UM runners had the highest body height and the heaviest body mass. Furthermore, M/UM runners were older. A study investigating master half-marathoners, master marathoners, and master ultra-marathoners found, however, no differences regarding their age, body mass, body height, and body mass index^36^. A study comparing recreational marathoners and recreational ultra-marathoners found differences in anthropometry where marathoners had a lower calf circumference but thicker skinfold thicknesses at pectoral, axilla, and suprailiacal sites compared to the ultra-marathoners^37^. Also, a study comparing recreational half-marathoners and marathoners reported that half-marathoners had a higher body mass, longer legs, a larger circumference of the upper arm, thicker thigh skinfolds, a higher sum of skinfold thicknesses, a higher body fat percentage, and a higher skeletal muscle mass than marathoners^29^. These disparate findings might be due to different sample sizes and performance levels of the subjects.

### Differences in motivation across groups

We found no difference across race distance subgroups regarding the motivation to compete or the associated motives (i.e., exercise motive, original motive to run, present motive to run, motive to race). Interestingly, this finding disagrees with previous findings^19,21,26^, and different aspects might explain the discrepancy. Methodological differences, including analysis stratification by sex and age groups^22,38^, training habits^39,40^, and country of residence^41^, can be related to the differences in the findings. Differences between the sexes were shown for marathoners, where women were more motivated about their weight, affiliation, psychological coping, life meaning, and self-esteem but were less driven by competition^38^. Ultra-marathoners presented higher scores on affiliation and life meaning and lower values for body weight concerns, personal goal achievement, and self-esteem^38,42^. The second running boom (1990s) increased the number of runners that are not aiming to become professional athletes but their engagement in competitions as a leisure/social activity^16^, which people used as a strategy to be involved in social groups as well as to know different places around the world^43^.

Furthermore, no significant difference was observed for the favorite race season. In elite marathoners, however, the seasonal distribution for marathon running has two peaks, spring (weeks 14 to 17) and autumn (weeks 41 to 44). During these two periods, the expected temperature is close to the optimal value for marathon running^44^. It is well-described that interrelationships between marathon results and weather factors such as air temperature, wet bulb temperature, and human biometeorological indices exist^45^. Most probably, recreational runners do not focus on environmental conditions but rather on a specific event they want to compete in.

### Differences in running experience across groups

We found a significant difference in the number of years of running completed (consecutively or inconsecutively) without taking a break, with M/UM runners reporting the highest number of years and 10 km runners reporting the lowest number. M/UM runners reported more years of fully active running experience and completed more of their planned marathon and ultra-marathon races in the previous two years compared to the 10 km runners. The higher time of experience for M/UM runners and more completed marathon and ultra-marathon races in the previous two years highlight the profile of this subgroup. Similar findings showed that long-distance runners were older than short-distance runners (i.e., 5 km, 10 km)^46,47^. These characteristics are also related to the age of peak performance since a positive relationship has been reported between the age of peak performance and the length of the race distance^48–50^. In this way, differences between the race distance subgroups regarding the best time performances can also be related to training background and running experience. Besides the genetic component^51^, the main physiological parameters associated with long-distance performance (i.e., maximal oxygen consumption (VO_2_max), running economy, lactate threshold, and velocity associated with VO_2_max) are developed during training through the increases in the mitochondrial content and skeletal muscle capillary density^32,52^. Besides that, marathon and ultra-marathon performance are strongly related to sex, morphological, and psychological variables^53,54^, which can act as confounders in the present study.

### Differences in previous performance across groups

We found significant differences between the race distance subgroups regarding the best time performances. On average, M/UM runners were faster to complete HM and M events. This finding is not in line with previous findings. Data covering 107.9 million race results, including 70,000 events held from 1986 to 2018, showed that non-professional marathoners were 18% and 17% slower compared to female and male half-marathoners, respectively^55^. In addition, the best performances can be related to the sex distribution among the subgroups since men are overrepresented in M/UM (62%). A body of evidence is available regarding running performance differences between sex^56,57^, where men tended to perform 10% better compared to women^56^. Data from previous research from the NURMI study confirms sex differences for years of active running, the number of races completed, and best time performance, with men being faster on average at HM and M distances compared to women^33^. However, these differences tended to be null when training routines, exercise habits, and racing history was considered confounders. These results indicate that regardless of the subgroup distance, training background is important for the best finish time, as shown previously^28^. In addition, when considering the confounders of running experience in years fully active in running without a break and the participants’ age at their first running event, M/UM runners remained the fastest on average to complete HM and M. These results highlight the multifactorial and complex nature of the cause of achieved results or successes in sports disciplines^58^.

### Differences in race performance across groups

We found that M/UM runners remained the fastest on average to complete HM and M events when considering the confounders of running experience in years fully active in running without a break and the participants’ age at their first running event. Thus, this exploratory investigation upholds the assumption that there is a difference in the best race performances considering time to finish between recreational endurance runners of different distances (10 km, HM, M/UM). A study comparing 10 km, half-marathon, and marathon showed differences regarding age and running speed between the groups^59^.

### Limitations

Considering the limitation of the cross-sectional design, this study’s findings have some limitations that should be addressed, including that no underlying causation can be acquired from the present results. The primary limitation for vital consideration is the self-report feature of the survey methodological approach, which is known to result in misrepresented answers due to social expectations^60^. In addition, study participation was voluntary, which may have led to a non-randomized study population, although the participants were highly motivated. For the present study, the distance groups were not stratified by sex, which limits the comparisons, and suggest that different sub-groups need to be studied among runners to better understand motives, routines, and physical exercise engagement. To limit the misreporting effect, the survey included control questions throughout the different parts. Additionally, highly motivated distance runners made up the study sample, which likely added to the reliability of their responses and enhanced the dataset. Moreover, the sample included 245 endurance runners, which was relatively small considering the commonality of running as a sport. Moreover, other individual (nutritional status or the nutritional type maintained by the participants) and environmental characteristics (the racing environment, and specific weather conditions) that affect training commitment and performance was not considered in the present study (but of the NURMI Study Step 3, not published so far). Despite this limitation, the present study presents some advances for the events organizations, coaches, and sports scientists to better understand amateur runners of different characteristics. In addition, the race distance subgroups were unequally distributed per se, considering that 37% of the total sample were 10 km runners, 36% were HM runners, and 27% were M/UM runners. Another limitation is that multiple aspects of running competitions were not controlled for, essentially the racing environment itself and the specific weather conditions (poor or good running weather, temperature, and humidity), the time of the event, the season, and competition region. Regardless, the best time performances were retrospectively verified under random selection. Lastly, the current investigation did not include nutritional status or the nutritional type maintained by the participants, as personal nutrition is well-known to affect performance. Even though this investigation did not include nutritional results, the NURMI study has obtained the runners nutritional evidence that was or will be published in other articles due to scientific journal publication demands.

## Conclusions

Runners competing in different race distances such as 10 km, half-marathon, marathon, and ultra-marathon presented differences in training background and habits according to the distance of preference. Marathoners and ultra-marathoners were older, taller, and heavier, were running for more years, and had faster personal best times than 10 km runners. Further studies need to consider the second level of information, considering the role of competition in runners' training commitment as well as environmental features related to training commitment.

## Data Availability

The data sets generated during and/or analyzed during the current study and presented in this article are not publicly available. Requests to access the datasets should be directed to info@nurmi-study.com. Subjects will receive a brief summary of the results of the NURMI Study if desired.

## References

[1] RunRepeat. *The State of Ultra Running 2020*. https://runrepeat.com/state-of-ultra-running. (2021).

[2] RunRepeat. *133 Stats on 5K Running Races in the US*. https://runrepeat.com/the-us-5k-stats-page (2021).

[3] RunRepeat. *Marathon Statistics 2019 Worldwide*. https://runrepeat.com/research-marathon-performance-across-nations (2020).

[4] Scheerder, J., Breedveld, K. & Borgers, J. *Running across Europe: The Rise and Size of One of the Largest Sport Markets* (Palgrave Macmillan, 2015).

[5] Kozlovskaia M (2019). A profile of health, lifestyle and training habits of 4720 Australian recreational runners: The case for promoting running for health benefits. Health Promot J. Austr..

[6] Parra-Camacho D, Alonso Dos Santos M, González-Serrano M (2020). Amateur Runners' Commitment: An analysis of sociodemographic and sports habit profiles. Int. J. Environ. Res. Public Health.

[7] Thuany M, Malchrowicz-Mośko E, Waśkiewicz Z, Gomes T (2021). Individual and economic characteristics as determinants of Brazilian runners’ motivation. Sustainability.

[8] Breuer C, Hallmann K, Wicker P (2013). Determinants of sport participation in different sports. Manag.

[9] Janssen M, Scheerder J, Thibaut E, Brombacher A, Vos S (2017). Who uses running apps and sports watches? Determinants and consumer profiles of event runners' usage of running-related smartphone applications and sports watches. PLoS ONE.

[10] Knechtle B (2021). Training and racing behavior of recreational runners by race distance-results from the NURMI Study (Step 1). Front. Physiol..

[11] Boldt P (2018). Quality of life of female and male vegetarian and vegan endurance runners compared to omnivores: Results from the NURMI study (step 2). J. Int. Soc. Sports Nutr..

[12] Wirnitzer K (2021). Sex differences in supplement intake in recreational endurance runners—results from the NURMI Study (Step 2). Nutrients.

[13] Wirnitzer K (2022). Who is running in the D-A-CH countries? An epidemiological approach of 2455 omnivorous, vegetarian, and vegan recreational runners-results from the NURMI Study (Step 1). Nutrients.

[14] Boldt P (2019). Sex Differences in the health status of endurance runners: Results from the NURMI Study (Step 2). J. Strength Cond. Res..

[15] Lee D-C (2014). Leisure-time running reduces all-cause and cardiovascular mortality risk. J. Am. Coll. Cardiol..

[16] Hautbois C, Djaballah M, Desbordes M (2019). The social impact of participative sporting events: A cluster analysis of marathon participants based on perceived benefits. Sport Soc..

[17] WHO. *Guidelines on Physical Activity and Sedentary Behaviour* (World Health Organization, 2020) https://www.who.int/publications/i/item/9789240015128.

[18] Malchrowicz-Mosko E, León-Guereño P, Tapia-Serrano M, Sánchez-Miguel P, Waśkiewicz Z (2020). What encourages physically inactive people to start running? An analysis of motivations to participate in parkrun and city trail in poland. Public Health Front..

[19] Whitehead A (2022). Motivational differences between 5K, half marathon and full marathon participants in the UK and India. Manag Sport Leisure.

[20] León-Guereño P, Galindo-Domínguez H, Balerdi-Eizmendi E, Rozmiarek M, Malchrowicz-Mośko E (2021). Motivation behind running among older adult runners. BMC Sports Sci. Med. Rehabil..

[21] Gerasimuk D (2021). Age-related differences in motivation of recreational runners, marathoners, and ultra-marathoners. Front. Psychol..

[22] Leon-Guereno P, Tapia-Serrano M, Castaneda-Babarro A, Malchrowicz-Mosko E (2020). Do sex, age, and marital status influence the motivations of amateur marathon runners? The Poznan Marathon Case Study. Front. Psychol..

[23] Manzano-Sánchez D, Postigo-Pérez L, Gómez-López M, Valero-Valenzuela A (2020). Study of the motivation of Spanish amateur runners based on training patterns and gender. Int. J. Environ. Res. Public Health.

[24] Waśkiewicz Z, Nikolaidis P, Chalabaev A, Rosemann T, Knechtle B (2019). Motivation in ultra-marathon runners. Psychol. Res. Behav. Manag..

[25] Stevinson C (2022). Adherence and health-related outcomes of beginner running programs: A 10-week observational study. Res. Q. Exerc. Sport.

[26] Rozmiarek M (2022). Motivation and eco-attitudes among night runners during the COVID-19 pandemic. Sustainability.

[27] León-Guereño P, Tapia-Serrano M, Sánchez-Miguel P (2020). The relationship of recreational runners’ motivation and resilience levels to the incidence of injury: A mediation model. PLoS ONE.

[28] Fokkema T (2020). Training for a (half-)marathon: Training volume and longest endurance run related to performance and running injuries. Scand. J. Med. Sci. Sports.

[29] Friedrich M (2014). A comparison of anthropometric and training characteristics between female and male half-marathoners and the relationship to race time. Asian J. Sports Med..

[30] Knechtle B, Knechtle P, Roseman T, Senn O (2010). Sex differences in association of race performance, skin-fold thicknesses, and training variables for recreational half-marathon runners. Percept. Motor Skills.

[31] Knechtle B (2022). Training habits in recreational half-marathon, marathon/ultra-marathon and 10-KM distance runners (Part A)-Results from the NURMI Study (Step 2). Sci. Rep..

[32] Tanous D (2022). Sex differences in training behaviors of 10 km to ultra-endurance runners (Part A)-results from the NURMI Study (Step 2). Int. J. Environ. Res. Public Health.

[33] Motevalli M (2022). Sex differences in racing history of recreational 10 km to ultra runners (Part B)-results from the NURMI Study (Step 2). Int. J. Environ. Res. Public Health.

[34] Wirnitzer K (2022). Health status of recreational runners over 10-km up to ultra-marathon distance based on data of the NURMI Study Step 2. Sci. Rep..

[35] Wirnitzer K (2016). Prevalence in running events and running performance of endurance runners following a vegetarian or vegan diet compared to non-vegetarian endurance runners: The NURMI Study. SpringerPlus.

[36] Knechtle B, Rüst CA, Knechtle P, Rosemann T (2012). Does muscle mass affect running times in male long-distance master runners?. Asian J. Sports Med..

[37] Rüst C, Knechtle B, Knechtle P, Rosemann T (2012). Similarities and differences in anthropometry and training between recreational male 100-km ultra-marathoners and marathoners. J. Sports Sci..

[38] Waskiewicz Z (2019). What motivates successful marathon runners? The role of sex, age, education, and training experience in polish runners. Front. Psychol..

[39] Krouse R, Ransdell L, Lucas S, Pritchard M (2011). Motivation, goal orientation, coaching, and training habits of women ultrarunners. J. Strength Cond. Res..

[40] Ogles B, Masters K (2000). Older vs. younger adult male marathon runners: participative motives and training habits. J. Sport Behav..

[41] Elbe A, Madsen C, Midtgaard J (2010). A cross-cultural comparison of motivational factors in Kenyan and Danish middle and long distance elite runners. J. Psychol. Afr..

[42] Doppelmayr M, Molkenthin A (2004). Motivation of participants in adventure ultramarathons compared to other foot races. Biol. Sport.

[43] Tian H, Qiu Y, Lin Y, Zhou W, Fan C (2020). The role of leisure satisfaction in serious leisure and subjective well-being: Evidence from Chinese Marathon Runners. Front. Psychol..

[44] Marca A (2014). Marathon progress: Demography, morphology and environment. J. Sports Sci..

[45] Zhang S, Meng G, Wang Y, Li J (1992). Study of the relationships between weather conditions and the marathon race, and of meteorotropic effects on distance runners. Int. J. Biometeorol..

[46] Thuany M, Gomes T, Rosemann T, Knechtle B, de Souza R (2021). No trends in the age of peak performance among the best half-marathoners and marathoners in the world between 1997–2020. Medicina.

[47] Nikolaidis P, Alvero-Cruz J, Villiger E, Rosemann T, Knechtle B (2019). The age-related performance decline in marathon running: The paradigm of the Berlin marathon. Int. J. Environ. Res. Public Health.

[48] Hoffman M (2010). Performance trends in 161-km ultramarathons. Int. J. Sports Med..

[49] Knechtle B, Nikolaidis P (2018). Physiology and Pathophysiology in Ultra-Marathon Running. Front. Physiol..

[50] de Souza R (2022). Ultramarathon evaluation above 180 km in relation to peak age and performance. BioMed Res. Int..

[51] Appel M, Zentgraf K, Krüger K, Alack K (2021). Effects of genetic variation on endurance performance, muscle strength, and injury susceptibility in sports: A systematic review. Front. Physiol..

[52] Holloszy JO (1967). Biochemical adaptations in muscle. Effects of exercise on mitochondrial oxygen uptake and respiratory enzyme activity in skeletal muscle. J. Biol. Chem..

[53] Buck K, Spittler J, Reed A, Khodaee M (2018). Psychological attributes of ultramarathoners. Wilderness Environ. Med..

[54] Knechtle B (2012). Ultramarathon runners: Nature or nurture?. Int. J. Sports Physiol. Perform..

[55] Andersen, J. J. *The State of Running 2019*. https://runrepeat.com/state-of-running (2019).

[56] Hallam L, Amorim F (2022). Expanding the gap: An updated look into sex differences in running performance. Front. Physiol..

[57] Besson T (2022). Sex differences in endurance running. Sports Med..

[58] Gajda R (2021). To be a champion of the 24-h ultramarathon race If not the heart _…_ mosaic theory?. Int. J. Environ. Res. Public Health.

[59] Nikolaidis P, Cuk I, Clemente-Suárez V, Villiger E, Knechtle B (2021). Number of finishers and performance of age group women and men in long-distance running: Comparison among 10km, half-marathon and marathon races in Oslo. Res. Sports Med..

[60] Althubaiti A (2016). Information bias in health research: Definition, pitfalls, and adjustment methods. J. Multidiscip. Healthc..

